# Understanding the path forward: a cross-sectional study of pathology trainee perspectives in the Republic of Ireland

**DOI:** 10.1007/s11845-026-04456-2

**Published:** 2026-05-23

**Authors:** Saied Ali, Zachary Ramsamaroo, Meadhbh Collison, Diarmuid Breathnach, Cynthia Heffron, Grace Chan, Maeve Doyle

**Affiliations:** 1 Department of Clinical Microbiology, Royal College of Surgeons in Ireland, Dublin, Ireland; 2 Department of Clinical Microbiology, University Hospital Waterford, Waterford, Ireland; 3 Department of Training, Education & Lifelong Learning, Royal College of Physicians of Ireland, Dublin, Ireland; 4 Department of Histopathology, Cork University Hospital, Cork, Ireland; 5 University College Cork, Cork, Ireland

**Keywords:** Chemical pathology, Clinical microbiology, Education, Haematology, Histopathology, Immunology, Pathology, Training

## Abstract

**Background:**

Pathology plays an essential role in diagnostics, guiding treatment decisions and medical research. In Ireland, a significant shortfall in the pathology workforce has emerged, driven by increasing service demands, population growth, and recruitment challenges. This study examines Irish trainee perspectives across pathology disciplines to identify factors influencing career choice and retention.

**Methodology:**

A cross-sectional survey was distributed to all Specialist Registrars registered with the Irish Faculty of Pathology, exploring self-reported perceptions of career motivations, training experiences, and future aspirations. Quantitative data were analysed descriptively, and qualitative comments were reviewed thematically.

**Results:**

Sixty trainees completed the survey (47.6% response rate). Interest in pathology most often developed during medical school (28.3%) or early postgraduate years (38.3%), with mentorship and practical exposure identified as key influences. Over half (58.3%) reported limited speciality-specific teaching during Basic Specialist Training and recommended expanding early teaching and rotations to strengthen early exposure. Flexibility was valued, with 58.3% preferring less-than-full-time training. Most respondents viewed pathology as stimulating and rewarding, recognising its contribution to patient care (91.5%) and good quality of life (77.5%). Career aspirations were diverse, with many expressing interests in clinical practice (65%), education (51.7%) and research (33.3%), reflecting the breadth of opportunities within the speciality.

**Conclusion:**

This survey highlights a motivated trainee cohort and identifies practical ways to strengthen pathology training in Ireland. Continued collaboration between trainees, training bodies, and institutions to enhance early exposure, teaching, mentorship, and flexible working will be vital to support recruitment and sustain a skilled and engaged workforce.

## Abstract (highlights)

**What is already known on this topic:**

Pathology is fundamental to diagnosis and clinical decision-making, yet workforce shortages are being reported in many health systems as demand for diagnostics increases. Studies from other countries suggest that career interest in pathology often depends on early exposure and mentorship, while limited visibility during clinical training may deter potential recruits. Recruitment and retention are also influenced by workload, work patterns, and changing expectations among younger doctors. In Ireland, workforce planning reports describe substantial consultant shortfalls, but little is known about how trainees themselves experience training or view their future careers.

**What this study adds:**

This study offers the first national overview of pathology trainee perspectives across specialties in Ireland. It shows that many trainees developed interest early in training, yet more than half reported limited specialty-specific teaching during basic training years. Participants described pathology as intellectually engaging and compatible with good quality of life, but highlighted staffing levels, workload, and on-call commitments as ongoing concerns. The strong interest in flexible working, education, and research points to a cohort seeking varied and sustainable career paths.

**How this study might affect research, practice or policy:**

The findings suggest that workforce planning should consider training experience, visibility, and working patterns alongside staffing numbers. Expanding structured exposure to pathology earlier in training may support recruitment, while attention to workload and flexibility may help retention. These results provide empirical evidence to inform training development and national workforce discussions in pathology.

## Introduction

Pathology is essential for diagnosing diseases, guiding treatment decisions and advancing medical research [1, 2]. It underpins more than 70% of clinical decisions [3, 4], from cancer detection and chronic disease monitoring to blood transfusion and outbreak management.

Despite this importance, Ireland is experiencing a pathology workforce crisis. At present, only 292 whole-time equivalent (WTE) consultant posts are filled, compared with an estimated national requirement of 466.6 WTEs to meet current population needs [5]. This shortage, exacerbated by rising demand for diagnostic services, poses serious challenges to healthcare delivery [6].

The “Medical Workforce Planning for the Specialities of Pathology – An Expert Stakeholder Informed Review (2023)” highlighted the urgency of intervention, recommending that Higher Specialist Training (HST) intake rise from 23 in 2022 to 70 by 2028. Yet, the demand for pathologists is expected to exceed supply, with an additional 191 consultants needed by 2035. This anticipated shortfall reflects not only population growth and longer life expectancy, but also the expanding scope of diagnostics driven by new technologies [5].

By 2035, Ireland’s population aged 61–85 is projected to increase by 40%, and those over 85 by 105% [7]. As the population ages, the incidence of cancer, cardiovascular disease and neurodegenerative disorders will increase, creating greater urgency for diagnostic testing. At the same time, advances in molecular and genomic technologies place pathologists at the forefront of precision medicine, while the coronavirus disease 2019 (COVID-19) underscored the necessity of resilient laboratory services [8].

Across Europe, postgraduate training in pathology has become more structured and standardised, guided by initiatives from the European Union of Medical Specialists (UEMS) [9]. The introduction of the European curriculum in Medical Microbiology in 2017, followed by the establishment of a pan-European examination, marked important steps towards a shared framework for specialist education [10]. A 2021 survey spanning 21 countries found that most had established national training programmes with defined curricula, accredited trainers, and formal assessments. Nonetheless, variation persisted, particularly in the duration of training and the balance between laboratory and clinical commitments [10]. A follow-up analysis in 2022 showed continued progress, with 95% of countries reporting a national training lead and almost three-quarters requiring an examination for specialist certification [11].

Within this broader European context, Ireland’s training pathway aligns closely with the UEMS framework. Pathology training in Ireland begins with Basic Specialist Training (BST), lasting two-to-three years, which provides a broad clinical foundation [12, 13]. Trainees then progress to HST, generally a five-year program focused on developing advanced clinical, diagnostic and leadership skills [12, 14]. These structured pathways, overseen by the Royal College of Physicians of Ireland (RCPI), reflect the competency-based and quality-assured principles set out in the European Training Requirements [12].

A broadly comparable pathway operates in the United Kingdom, where trainees enter specialty-specific pathology training at Specialty Training year 1 (ST1) following completion of the two-year Foundation Programme and, for most specialties, a period of core training. From the point of entry into specialty training, overall training duration, and assessment requirements, including a mandatory exit examination, are broadly similar between the two systems. The principal distinction lies in training delivery. Pathology training in the UK is organised through regionally-based programmes under the oversight of the Royal College of Pathologists (RCPath), whereas Ireland’s HST model is nationally coordinated, with structured rotations across multiple centres, reflecting many of the core aims of UEMS [15].

Ireland’s six pathology specialities – chemical pathology, clinical microbiology, haematology, histopathology, immunology, and neuropathology – are entering a period of significant change and opportunity. Advances in diagnostic technology and molecular medicine are expanding the scope of each discipline and strengthening their contribution to personalised healthcare. In chemical pathology, test volumes are increasing by 4.4% per year, driven by developments in point-of-care testing and new analytical platforms. Haematology is increasingly integrated into oncology and transfusion medicine, while histopathology remains pivotal to cancer screening, biomarker validation, and tissue-based diagnostics. Clinical microbiology remains essential to infection prevention and control, antimicrobial stewardship and the global response to antimicrobial resistance, strengthened by genomic surveillance systems that proved vital during the COVID-19 pandemic [16]. Similarly, immunology and neuropathology are playing expanding roles in the diagnosis and management of immune-mediated and neurodegenerative diseases, areas advancing rapidly with the arrival of novel therapeutic approaches [5].

Rather than suggesting a burden, this growing complexity highlights the increasing importance of pathology within multidisciplinary care. Internationally, the role of the pathologist has evolved well beyond traditional morphological interpretation to one of active clinical partnership [17, 18]. These developments are shaping pathology into a dynamic and intellectually stimulating field, marked by expanding clinical collaboration and new opportunities for leadership, innovation and translational research [19].

Nonetheless, pathology continues to face challenges with recruitment and retention, shaped by wider systemic factors [20]. These pressures are not confined to pathology but reflect broader trends across the medical workforce. The “Health Service Executive Medical Recruitment and Retention Report (2024)” highlights that almost all medical specialities experience difficulties in sustaining consultant numbers, influenced by changing career expectations and global mobility [21]. Similarly, the World Health Organization (WHO) anticipates a shortfall of up to ten million healthcare workers by 2030, emphasising that workforce shortages are structural in nature and extend well beyond any single discipline [22].

Within this wider context, the challenges facing pathology can also be seen as opportunities for growth. Advances in diagnostics and technology have expanded the speciality’s clinical reach, creating new roles for pathologists with skills in bioinformatics and molecular medicine. Rather than reinforcing old stereotypes of limited patient contact, these changes are strengthening pathology’s contribution to personalised care and multidisciplinary practice [23, 24].

Tackling workforce pressures will nonetheless require a multi-faceted approach, informed by the experiences of those already working in the system. Our study aims to address these challenges by exploring the expectations, motivations, and experiences among current trainees in the Republic of Ireland toward their respective pathology specialities. By understanding what drives individuals to pursue pathology and identifying barriers that deter others, can help inform practical strategies to improve recruitment and retention.

## Materials and methods

A cross-sectional study was undertaken to explore the expectations, motivations, and experiences of current Specialist Registrars within the Irish Faculty of Pathology. The study population included all trainees registered with the RCPI across the six pathology disciplines – chemical pathology, clinical microbiology, haematology, histopathology, immunology, and neuropathology. At the time of data collection, this cohort comprised three trainees in chemical pathology, thirty-seven in clinical microbiology, thirty-six in haematology, forty in histopathology, eight in immunology and two in neuropathology [5].

An electronic survey was distributed via RCPI between 10th June and 8th July 2024. Demographic information, including age, gender and speciality, was collected to contextualize findings. The survey explored perceived strengths, and weaknesses of each discipline, and how trainees believe pathology is viewed by peers and within the wider healthcare system, along with factors influencing speciality choice. It also explored participants’ career aspirations, including preferred practice settings, anticipated future roles and interest in subspecialisation. Finally, respondents were invited to highlight perceived gaps in training and to suggest ways training within their discipline could be improved.

The survey included a combination of closed and open-ended questions. Closed questions used structured response options, including ranking and a 5-point Likert scale ranging from 1 (strongly disagree) to 5 (strongly agree). Open-ended questions provided space for free-text comments, enabling participants to elaborate on their experiences and perspectives.

All responses were anonymised before analysis. Quantitative data was summarised using descriptive statistics, including means, weighted means, frequencies, and standard deviations (σ). No inferential statistical analyses were planned or performed, as the study was designed to provide a descriptive overview of trainee perspectives. Qualitative data was analysed thematically to identify patterns as well as distinctive viewpoints. As participation was voluntary, the potential for self-selection bias is acknowledged.

## Results

### Demographic data

**Fig. 1:**
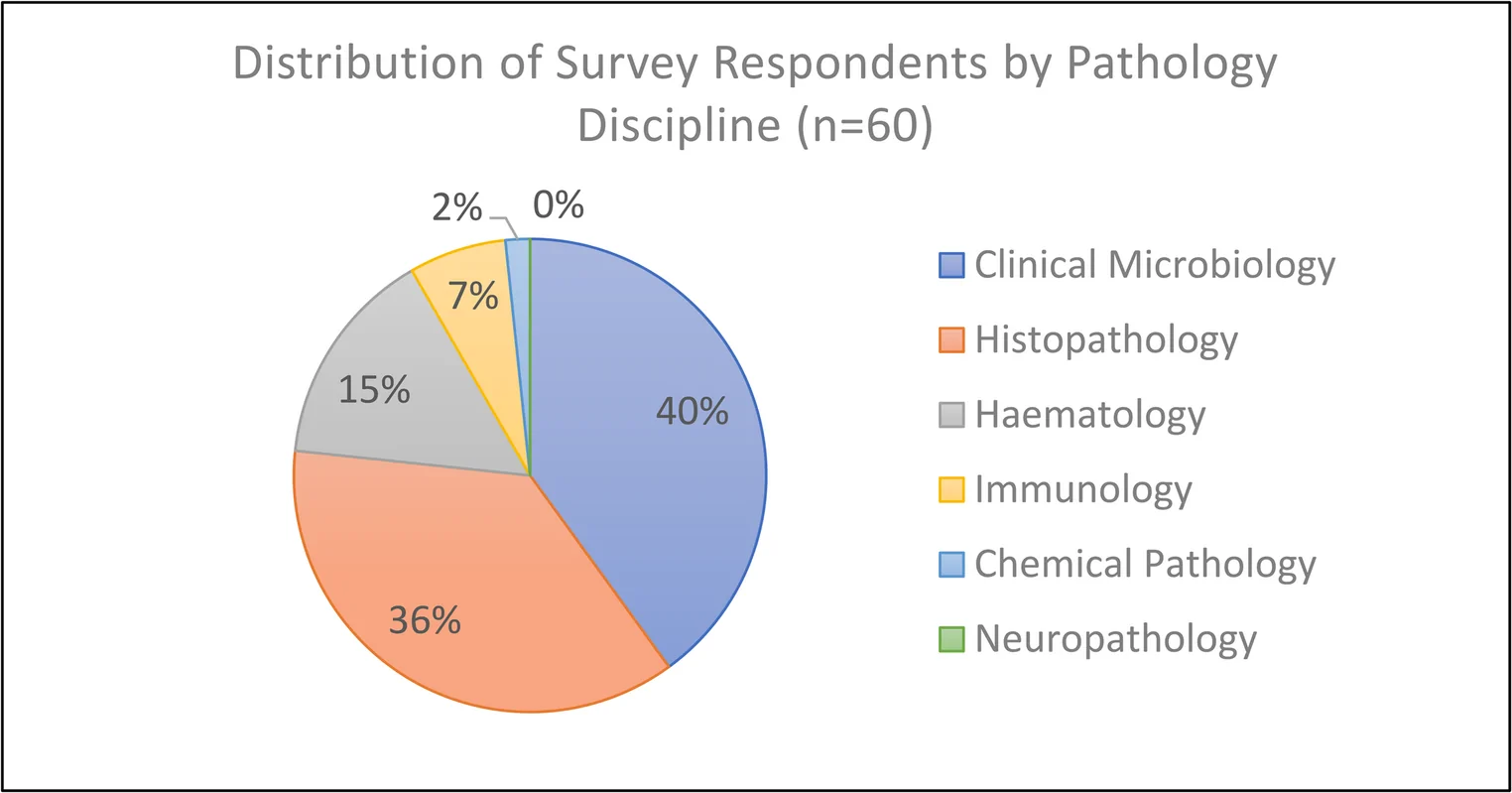
Distribution of respondents across pathology disciplines

Of the 126 Specialist Registrars invited, sixty completed the survey, giving an overall response rate of 47.6% across pathology disciplines. Respondents included twenty-four trainees in clinical microbiology, twenty-two in histopathology, nine in haematology, four in immunology and one in chemical pathology, while no responses were received from neuropathology (Fig. 1).

### Interest, motivation and speciality exposure

Just over one-quarter of respondents (28.3%, *n* = 17) first developed an interest in pathology during medical school, while more than one-third (38.3%, *n* = 23) identified their senior house officer years as the key period in developing that interest. Overall, 60% (*n* = 36) reported deciding to pursue a career in pathology during these early training periods. Mentorship strongly influenced career choice, with two-thirds (66.7%) citing consultants and over half (53.3%) citing registrars as key motivators. These trends were consistent across all pathology disciplines.

Many trainees had undertaken preparatory activities before entering their speciality, with 65% participating in audits and just over half (51.7%) completing clinical rotations or attachments in pathology or related fields.

Opportunities for postgraduate teaching during Basic Specialist Training (BST) showed notable variation across disciplines. More than half of respondents (58.3%) reported receiving no formal or structured teaching specifically related to their current pathology speciality during their BST years. This was most evident among clinical microbiology trainees, where 70.8% reported limited speciality-specific teaching.

### Perceptions of pathology

**Fig. 2:**
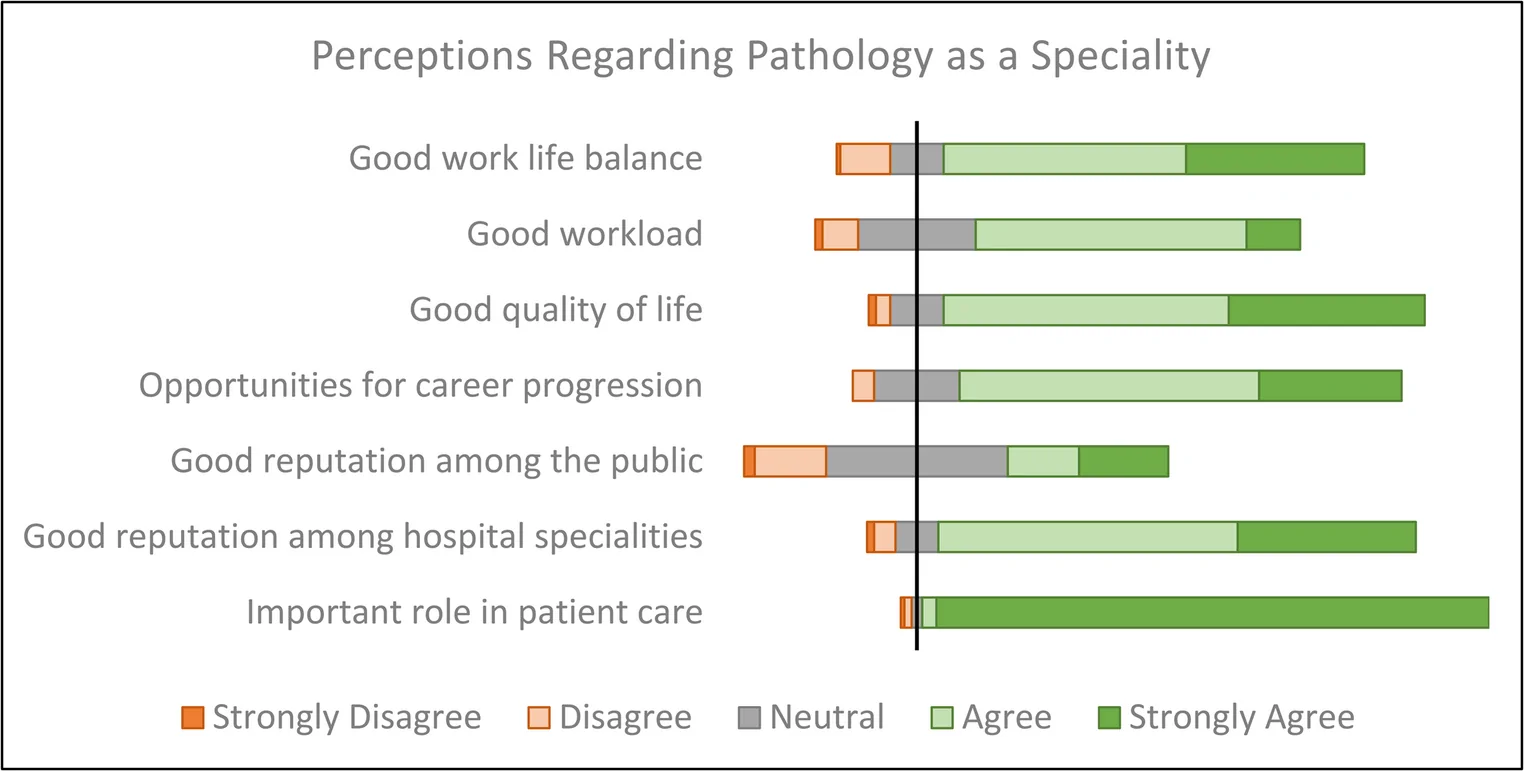
Perceptions of pathology among trainees

Perceptions of pathology across several domains are presented in Fig. 2. Almost all respondents (91.5%) agreed or strongly agreed that pathology plays an important role in patient care. A majority (77.5%) also rated its reputation among hospital specialities favourably. In contrast, responses were more divided regarding pathology’s reputation among the public. When asked to what extent they agreed that pathology has a good reputation among the public, one quarter (25%) agreed or strongly agreed, 42.5% were neutral, and 32.5% disagreed or strongly disagreed. Most respondents agreed that pathology offers opportunities for career progression (72.5%), good quality of life (77.5%), and good work–life balance (67.5%).

### Future career plans

**Fig. 3:**
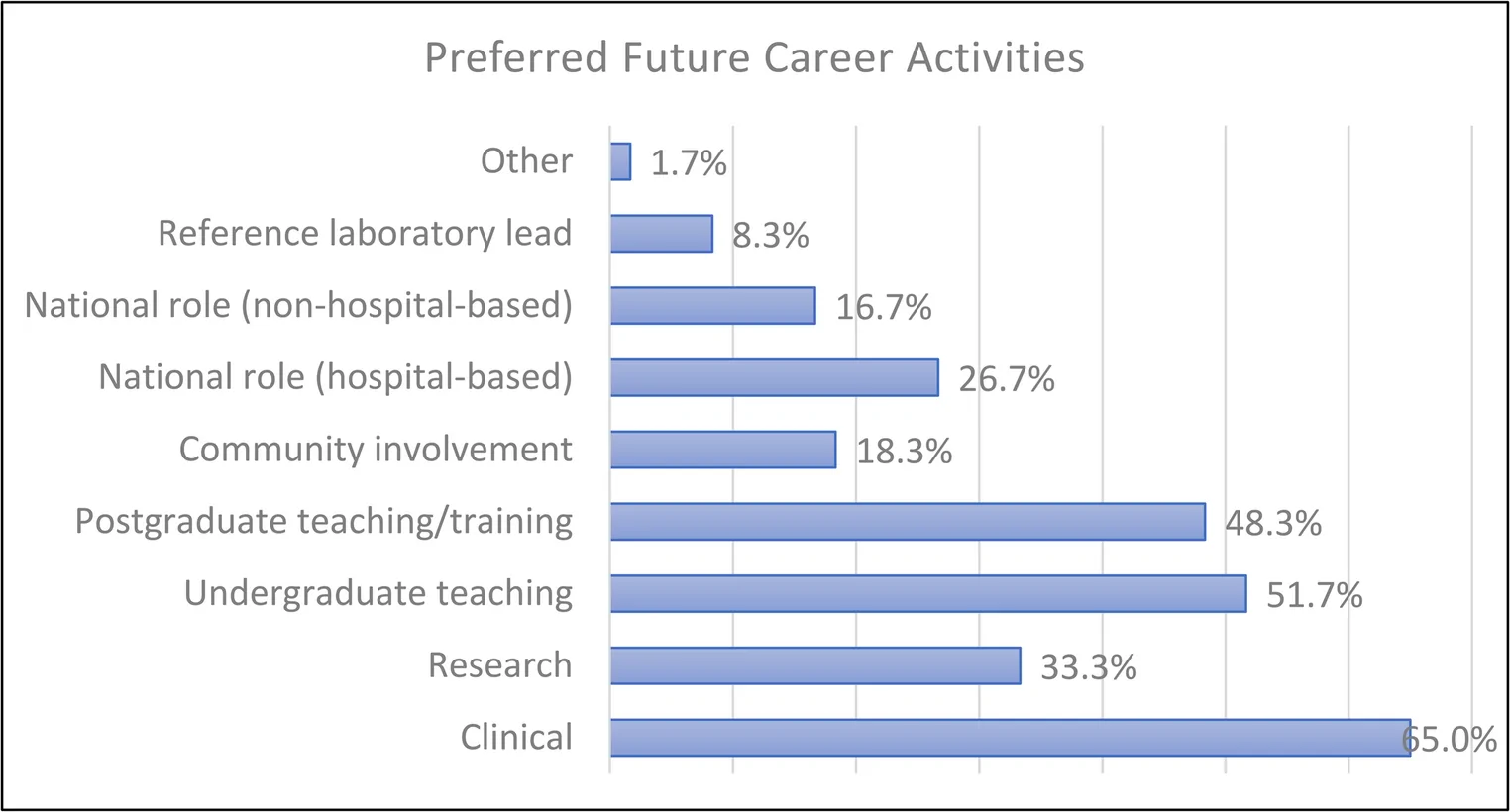
Overall preferred future career activities across all pathology disciplines

Diverse career aspirations were reported among respondents (Fig. 3; Table 1). Most trainees (65%) expressed interest in clinical work involving direct patient care or acute clinical decision-making. This preference was universal among chemical pathology and immunology trainees (100%) and was also prominent in haematology (77.8%) and clinical microbiology (62.5%). Research was identified as a goal by one-third of respondents (33.3%), with the highest interest among immunology (75%) and haematology (55.6%) trainees. Teaching featured strongly across disciplines, with more than half of respondents (51.7%) expressing an interest in undergraduate education and 48.3% in postgraduate teaching or training. This interest was especially noted among chemical pathology, clinical microbiology, and haematology trainees. National leadership roles were also viewed positively, with 26.7% interested in hospital-based and 16.7% in non-hospital-based positions, particularly among histopathology, immunology, and clinical microbiology trainees. Collectively, these findings reflect a workforce motivated by a combination of clinical engagement, academic contribution, and leadership development.

**Table 1:** Future career activities and workplace preferences across each pathology discipline

	Chemical Pathology (*n* = 1)	Clinical Microbiology (*n* = 24)	Haematology (*n* = 9)	Histopathology (*n* = 22)	Immunology (*n* = 4)
	*n* (%)	*n* (%)	*n* (%)	*n* (%)	*n* (%)
Which activities would you like to pursue in your future career?					
Clinical	1 (100%)	15 (62.5%)	7 (77.8%)	12 (54.5%)	4 (100%)
Research	0	8 (33.3%)	5 (55.6%)	4 (18.2%)	3 (75%)
Undergraduate teaching	0	14 (58.3%)	6 (66.7%)	10 (45.5%)	2 (50%)
Postgraduate teaching	1 (100%)	16 (66.7%)	6 (66.7%)	9 (40.9%)	1 (25%)
Community Involvement	1 (100%)	9 (37.5%)	0	1 (4.5%)	0
National role (hospital-based)	0	4 (16.7%)	2 (22.2%)	7 (31.8%)	2 (50%)
National role (non-hospital based)	1 (100%)	6 (25%)	2 (22.2%)	2 (9.1%)	0
Reference laboratory	0	4 (16.7%)	1 (11.1%)	0	0
Where would you like to work on completion of specialist training?					
Public/tertiary hospital (Model 4)	1 (100%)	15 (62.5%)	6 (66.7%)	15 (68.2%)	3 (75%)
Public/secondary hospital (Model 3)	0	9 (37.5%)	3 (33.3%)	6 (27.3%)	0
Public/primary (Model 2)	0	4 (16.7%)	1 (11.1%)	2 (9.1%)	0
Public/paediatric hospital	1 (100%)	3 (12.5%)	0	1 (4.5%)	0
Public/maternity hospital	0	2 (8.3%)	0	1 (4.5%)	0
Public/specialist hospital	0	5 (20.8%)	0	2 (9.1%)	1 (25%)
Private hospital	0	4 (16.7%)	0	4 (18.2%)	1 (25%)
Community health	0	8 (33.3%)	0	1 (4.5%)	0
Governmental organisation	0	5 (20.8%)	1 (11.1%)	4 (18.2%)	0
Research institute	0	4 (16.7%)	0	3 (13.6%)	1 (25%)

Workplace preferences varied across respondents. Two-thirds (66.7%) indicated a preference for tertiary public hospitals (Model 4), while 30% favoured secondary public hospitals (Model 3). Smaller numbers expressed interest in private hospitals (15%), community health settings (13.3%) and governmental organisations (16.7%). Research institutes and other public hospital types, such as paediatric, maternity and specialist hospitals, were less commonly selected. These patterns largely reflect how pathology services in Ireland are currently centred within major tertiary hospitals. Discipline-specific distributions are shown in Table 1.

Overall, 58.3% of respondents expressed a preference for working less than full-time but more than half-time. This preference was most common among clinical microbiology trainees (79.2%), while haematology trainees more often indicated a preference for full-time work. Many respondents valued flexible working arrangements, highlighting their importance in balancing research, academic activity, and family commitments.

Trainees were asked to identify areas of professional interest within their pathology discipline. Interests varied across specialties, although all reported strong engagement with molecular diagnostics and laboratory management. Clinical microbiology trainees most frequently selected bacteriology and antimicrobial stewardship, while haematology trainees highlighted both benign and malignant haematological conditions. Histopathology trainees prioritised solid organ tumours and molecular pathology, whereas immunology trainees most often identified primary immunodeficiencies and allergy. Chemical pathology trainees reported genetic and metabolic diseases as their main areas of focus. Collectively, these patterns reflect the broad scientific and clinical scope of pathology and its increasing alignment with molecular and precision medicine.

### Measures to increase attractiveness of pathology discipline

Preferences for measures to improve the attractiveness of pathology are presented in Table 2.

**Table 2:** Trainees’ opinions on how the attractiveness of their pathology discipline can be improved

	Weighted Averages Based on a 5-Point Likert Scale						Inter-Speciality σ
	Overall	Chemical Pathology	Clinical Microbiology	Haematology	Histopathology	Immunology	
Improve teaching/exposure in medical school	4.075	4	4.27	3.50	4.07	4.25	0.31
Decrease workload	3.6	2	3.60	4.50	3.36	2.75	0.94
Decrease on-call commitments	3.475	3	4.07	4.33	2.86	2.25	0.87
Facilitate flexible training and working contracts	4.25	3	4.53	4.17	4.21	3.75	0.59
Increase availability of academic positions	3.65	3	3.80	3.67	3.29	4.5	0.57
Increase staffing	4.5	5	4.27	4.50	4.57	5	0.32
Increase opportunities in the private sector	2.825	3	2.83	2.83	2.50	3.5	0.37
Increase opportunities in NGOs, governmental and intergovernmental organisations	3.275	3	3.47	2.67	3.21	3.75	0.42
Develop the international network	3.675	5	3.67	3.33	3.57	4.5	0.71
Diversify training fields	3.575	5	3.53	3.17	3.64	4	0.70
Increase face-to-face consultations and ward presence	3.05	5	3.73	2.33	2.71	2.5	1.12
Increase participation in MDTs	3.575	5	4.13	2.33	3.71	2.75	1.07
Offer an in-patient service	2.6	3	2.47	2.83	2.64	2.5	0.23
Facilitate BST rotations	4.05	2	4.40	4.17	3.57	4.75	1.08
Increase practical laboratory work	3.4	4	3.67	3.67	2.79	4	0.50

Expanding the pathology workforce was identified as the top priority across all disciplines (overall weighted average 4.5; σ = 0.32). Many trainees linked this to the need for a more balanced distribution of work and more sustainable on-call arrangements (overall σ = 0.94 and 0.87, respectively), recognising that increasing staffing levels could help ease service pressures and enhance trainee experiences.

Early exposure to pathology was also highlighted as a key area for improvement. In addition to the existing histopathology BST programme, trainees supported expanding pathology-specific BST rotations across other disciplines (overall weighted average 4.05; σ = 1.08) and increase students’ exposure to medical pathology as a career through pathology teaching in medical school (overall weighted average 4.08; σ = 0.31). Together, these measures were viewed as important steps in strengthening early engagement and recruitment into the speciality.

Differences across disciplines reflected the varied ways pathologists contribute to patient-centred care. Some specialities, such as clinical microbiology and chemical pathology, placed greater value on direct clinical interaction through face-to-face consultations and multidisciplinary team (MDT) meetings, reflecting their close collaboration with clinical teams in real-time decision-making. Others, including haematology and immunology, emphasised different forms of patient engagement, such as laboratory interpretation, diagnostic guidance, and integration of complex results into treatment plans.

Across all disciplines, practical laboratory work remained moderately important (2.79–4.00; σ = 0.50), but trainees also recognised that modern pathology increasingly extends beyond the bench, encompassing leadership, management, and strategic roles within healthcare systems. Moderate variation in the importance of developing international networks (σ = 0.71) and diversifying training fields (σ = 0.70) further reflected this evolving landscape, with many trainees viewing such opportunities as essential for broadening their impact and shaping the future direction of the speciality.

## Discussion

This survey explored the perspectives of pathology trainees across Ireland and provided valuable insight into how they experience and perceive their speciality. The findings reveal both strengths and challenges within current training, highlighting a workforce that is deeply engaged and motivated.

Interest in the speciality most commonly developed during medical school (28.3%) or in the early postgraduate years as senior house officers (38.3%), a pattern consistent with reports in the literature [23, 25]. Respondents recognised these formative periods and proposed an earlier, more structured exposure in BST and during undergraduate training to improve recruitment. More than half (58.3%) reported receiving no formal speciality-specific teaching in their discipline during BST and identified this as a key area for improvement. Many also recommended expanding pathology-specific BST rotations beyond histopathology to provide earlier exposure across all disciplines. Encouragingly, many trainees also expressed a personal commitment to change this narrative, with 51.7% interested in undergraduate teaching and 48.3% in postgraduate education. Pathology registrars also now participate in regular peer-to-peer tutorials and are increasingly visible in leadership roles such as lead non-consultant hospital doctors (NCHDs). This willingness to contribute to teaching reflects a growing sense of ownership among trainees in shaping the future of the speciality.

Furthermore, trainees highlighted reduced visibility of pathology during the clinical years of medical school, coinciding with a greater emphasis on patient facing specialties. As a result, opportunities to observe the integration of laboratory findings into clinical decision making may be limited [26–29]. Those who had more hands-on exposure through audits (65%) or attachments and rotations (51.7%) tended to develop an interest earlier [30]. These experiences make the discipline visible and relevant, showing its impact on diagnosis and treatment and underscoring the value of early, practical engagement in sustaining interest.

The RCPI and Faculty of Pathology have actively engaged with trainee feedback, incorporating these perspectives into programme development [31]. In parallel, universities and affiliated clinical sites increasingly promote audits, observerships and elective placements, reflecting a collaborative approach to supporting future pathologists.

Mentorship featured strongly, with 66.7% of respondents identifying consultants and 53.3% naming registrars as key motivators. The RCPI supports structured mentorship schemes alongside train the trainer programmes, linking senior and junior trainees across BST and HST and strengthening supervisory and professional development [32, 33]. This renewed focus on mentorship has the potential to attract new trainees and to challenge long-standing perceptions of pathology as impersonal.

Flexibility is an increasingly recognised strength of pathology training in Ireland, with 58.3% of respondents preferring less-than-full-time training to balance clinical work with research, teaching, or family life. This priority is reflected in the RCPI 2025–2029 strategic pillars, which emphasise flexibility and wellbeing as central to workforce planning, alongside consistent institutional support for less-than-full-time training [34].

Pathology was described by many trainees as an intellectually engaging and rewarding speciality. Most agreed that it offers a good quality of life (77.5%), clear opportunities for progression (72.5%), and moreover, a meaningful contribution to patient care (91.5%). These perspectives reflect the wide variety of roles available within the field, spanning diagnostics, teaching, research, innovation, and leadership. National initiatives such as the all-Ireland Irish Clinical Academic Training (ICAT) and Irish Clinician Educator Training (ICET) programmes provide valuable pathways for those pursuing combined academic and clinical careers [35, 36]. By 2024, nine pathology trainees had been appointed to ICAT fellowships, reflecting a growing presence of the speciality within Ireland’s clinician-scientist community.

Although pathology’s role in patient care is well recognised within hospitals, only 25% of respondents felt that it has a good reputation among the public. This gap highlights the ongoing need for advocacy and engagement beyond the laboratory [2, 37].

RCPI initiatives, including International Pathology Day and the annual St Luke’s Symposium, incorporate patient and public involvement (PPI) and aim to improve the visibility and understanding of pathology [38]. Irish pathologists also engage in international conferences and public forums, contributing to broader professional and public discourse on the role of pathology in healthcare [39, 40].

Differences between disciplines were clear in how trainees thought pathology could be made more attractive. Clinical microbiology and chemical pathology trainees placed greater value on face-to-face consultation and participation in MDT meetings, while haematology, histopathology and immunology trainees emphasised diagnostic integration and molecular expertise. Together, these patterns reflect the different ways pathology delivers patient-centred care, from direct clinical engagement to laboratory interpretation and translational research. Some respondents, particularly in clinical microbiology, also expressed interest in community health and public health collaboration, reflecting how the evolving goals of Sláintecare are broadening opportunities for pathology to contribute to integrated models of care in Ireland [41]. This breadth is a strength of the speciality, creating multiple routes into patient care, leadership and innovation [17, 42, 43].

Findings from this survey should be interpreted within the context of wider international workforce pressures. Evidence from the UK illustrates the potential consequences of sustained staffing shortages, with the 2025 RCPath Workforce Census reporting limited time for professional development, high workload burden and frequent stress among consultant pathologists [44]. While the present study did not directly assess burnout, trainee concerns regarding workload and the strong prioritisation of increased staffing, flexibility and sustainable working patterns suggest early indicators of similar pressures within the Irish system. These findings highlight the importance of engaging with trainee perspectives at this stage, as failure to address workforce capacity and training sustainability risks replicating challenges observed elsewhere [39, 44, 45].

This study has several limitations. As a cross-sectional survey, responses reflect perceptions at a single time point and do not permit causal inference or assessment of change over time. Participation was voluntary, introducing the potential for self-selection bias, as more engaged trainees may have been more likely to respond. Representation across subspecialties was uneven, limiting discipline-specific generalisability. Findings are based on self-reported perceptions rather than objective measures of training experience or career progression. The absence of longitudinal or outcome data also precludes evaluation of how reported experiences or intentions translate into future career trajectories.

Nonetheless, findings portray a speciality that is dynamic, collaborative, and open to progress. Pathology training in Ireland continues to evolve through ongoing conversations between trainees, trainers, and professional bodies. With continued investment and engagement, pathology is well placed to remain a vibrant and rewarding career that links science with patient care.

## Conclusion

This study presents a national overview of the perspectives of pathology trainees in Ireland, highlighting both the progress made and the challenges that remain. Interest in pathology most often developed during medical school or early postgraduate years, and trainees identified the need for earlier, structured exposure to strengthen recruitment. Mentorship, teaching, and flexibility were consistent priorities, with many expressing interests in educational roles and valuing the growing support for less-than-full-time training.

The RCPI and Faculty of Pathology have shown genuine responsiveness to trainee feedback, expanding mentorship schemes, promoting leadership opportunities, and embedding wellbeing into workforce planning. Pathology was widely described as stimulating and rewarding, with trainees recognising its vital contribution to patient care and its expanding scope across research, diagnostics, and innovation.

With continued investment in training, visibility, and workforce development, pathology in Ireland is well placed to remain a dynamic and fulfilling speciality.
